# Use of Artificial Intelligence-Assisted Histopathology for Evaluation of Sex-Specific Progression and Regression of Hepatocellular Carcinoma Related to Metabolic Dysfunction-Associated Fatty Liver Disease

**DOI:** 10.3390/diagnostics16020234

**Published:** 2026-01-11

**Authors:** Ke Yin, Yuyun Song, Ran Fei, Xu Cong, Baiyi Liu, Zilong Wang, Xin Ai, Minjun Liao, Yayun Ren, Kutbuddin Akbary, Wei Wang, Qiang Yang, Xiao Teng, Nan Wu, Huiying Rao, Xiaoxiao Wang, Feng Liu

**Affiliations:** 1 Peking University People’s Hospital, Peking University Hepatology Institute, Infectious Disease and Hepatology Center of Peking University People’s Hospital, Beijing Key Laboratory of Hepatitis C and Immunotherapy for Liver Diseases, Beijing International Cooperation Base for Science and Technology on NAFLD Diagnosis, Beijing 100044, China; yinke_iris@sina.cn (K.Y.); songyy199802@outlook.com (Y.S.); lawpha@sina.com (R.F.); congxu@pkuph.edu.cn (X.C.); liubaiyi666@163.com (B.L.); zeelomwang@bjmu.edu.cn (Z.W.); 2311110382@stu.pku.edu.cn (X.A.); minjunliao189@163.com (M.L.); wn_summer@163.com (N.W.); raohuiying@pkuph.edu.cn (H.R.); 2 HistoIndex Pte Ltd., Singapore 117674, Singapore; ren.yayun@choututech.com (Y.R.); akbary.kutbuddin@histoindex.com (K.A.); teng.xiao@choututech.com (X.T.); 3 Hangzhou Choutu Technology Co., Ltd., HangZhou 310052, China; wang.wei@choututech.com (W.W.); yang.qiang@choututech.com (Q.Y.)

**Keywords:** metabolic dysfunction-associated fatty liver disease, hepatocellular carcinoma, animal model, female, male

## Abstract

**Background/Objectives:** Sex-specific differences in metabolic dysfunction-associated fatty liver disease (MAFLD)-related hepatocellular carcinoma (HCC) remain poorly understood. This study aimed to clarify sex-associated disparities in disease progression and recovery using a diethylnitrosamine (DEN) plus Western diet/fructose-induced murine model combined with artificial intelligence (AI)-assisted histological analysis. **Methods:** Male and female C57BL/6J mice received a single diethylnitrosamine injection and were fed a Western diet/fructose regimen for 38 weeks, followed by an 8-week recovery period on standard chow. Serum biochemical parameters were measured, and liver histology was assessed using second harmonic generation/two-photon excitation fluorescence (SHG/TPEF) microscopy. Steatosis and fibrosis were quantified within tumor and adjacent non-tumor regions using AI-based image analysis. **Results:** Male mice developed more severe disease phenotypes, including greater tumor burden and higher serum alanine aminotransferase levels, compared with females. Following dietary recovery, female mice showed substantial reductions in tumor number and hepatic steatosis, particularly in non-tumor regions; in contrast, male mice demonstrated only minimal improvement. AI-assisted quantification confirmed considerable regression of both steatosis and fibrosis in females and moderate fibrosis improvement in both sexes. **Conclusions:** These findings indicate sexual dimorphism in the progression and regression of MAFLD-related HCC, with females exhibiting enhanced metabolic and histological recovery. The results underscore the importance of considering sex as a biological variable in preclinical metabolic dysfunction–associated fatty liver disease-related hepatocellular carcinoma research and highlight the value of AI-enhanced imaging for precise, objective evaluation of liver histology.

## 1. Introduction

Metabolic dysfunction-associated fatty liver disease (MAFLD) is a chronic liver condition driven by overnutrition and insulin resistance, and its prevalence continues to rise in parallel with global increases in obesity and type 2 diabetes mellitus [1]. Recent epidemiological data estimate a global prevalence of 32.4% and a prevalence of 29.6% in China [2,3]. MAFLD is now recognized as a leading cause of cirrhosis and hepatocellular carcinoma (HCC). Disease progression typically advances from a healthy liver to MAFLD, metabolic dysfunction-associated steatohepatitis (MASH), and fibrosis, ultimately leading to cirrhosis and HCC [4]. From 2022 to 2050, the proportion of MASH-related HCC is projected to increase from 8% to 10.8% [5]. Therefore, controlling metabolic risk factors is essential to reducing the burden of MAFLD-related HCC (MAFLD-HCC). Recently, the FDA approved Resmetirom and Wegovy for the treatment of MASH [6,7]; however, further research is needed to clarify the mechanisms underlying MAFLD-HCC and to develop effective therapeutic strategies.

Growing evidence highlights the importance of sexual dimorphism in MAFLD progression [8]. Epidemiological studies consistently report a higher prevalence and severity of MAFLD and HCC in men and postmenopausal women compared with premenopausal women, suggesting a protective role of estrogen [9]. Despite this, most preclinical studies investigating MAFLD pathogenesis and treatment rely primarily on male rodent models. This imbalance has limited our understanding of female-specific disease characteristics and hormonal influences on both progression and, critically, regression, potentially overlooking opportunities for sex-specific therapeutic strategies.

Since ethical and logistical limitations constrain clinical trials, animal models are widely used to study MAFLD mechanisms and treatments. Several models [10,11,12,13,14], including high-fat and fructose diets combined with diethylnitrosamine (DEN) or MUP-uPA transgenic mice, recapitulate key clinical features such as inflammation, fibrosis, and tumorigenesis. However, despite well-established sex differences, systemic comparisons of disease progression and regression between males and females in these models remain limited.

To address this gap, we focused on histopathological differences between male and female mice during both progression and regression of DEN plus Western diet (WD)-induced MAFLD-HCC. Given that artificial intelligence (AI) has been increasingly utilized in pathological analysis [15,16,17,18,19], we utilized second harmonic generation/two-photon excitation fluorescence (SHG/TPEF) microscopy combined with AI image analysis to quantitatively assess steatosis and fibrosis in unstained liver tissues. This approach provides objective and automated quantification, overcoming the limitations of traditional semi-quantitative scoring.

We aimed to clarify sex-associated disparities in disease progression and recovery with AI-assisted histological analysis and generate a robust evidence base to guide appropriate model selection in future studies and to contribute to the development of sex-specific diagnostic and therapeutic strategies for MAFLD-HCC.

## 2. Materials and Methods

### 2.1. MAFLD-HCC Mouse Models

Male C57BL/6J mice were obtained from Beijing HFK Bioscience Co., Ltd. (Beijing, China). Animals were housed at 25 °C under a 12-h light/dark cycle and were provided standard chow and water ad libitum until experimental procedures began. All animal protocols were approved by the Animal Experimental Ethics Committee of Peking University People’s Hospital (No. 2021PHE111). A WD (21.2% fat, 17.3% protein, and 48.5% carbohydrates; TD120528) was purchased from Medicine Ltd. (Yangzhou, China), and a standard chow diet (SCD) was supplied by SPF Biotechnology Co., Ltd. (Beijing, China). DEN (Sigma-Aldrich, cat#: N0756, St. Louis, MO, USA) was dissolved in 0.9% saline immediately before use.

To induce HCC, 3-week-old C57BL/6J mice received a single intraperitoneal injection of DEN. At 6 weeks of age, mice were randomly assigned by sex to one of two groups:

1. Group 1 (38 weeks): Mice were fed WD and sacrificed at 38 weeks of age.
2. Group 2 (46-week regression): Mice received WD until 38 weeks of age, were then switched to a standard chow diet for 8 weeks, and sacrificed at 46 weeks of age.

Baseline control mice (six males and six females) were evaluated at 6 weeks of age. Body weight and general health were assessed every 4 weeks. Six mice per group were euthanized at each designated time point.

### 2.2. Blood Assessment

Blood samples were collected at the specified time points to measure serum alanine aminotransferase (ALT), aspartate aminotransferase (AST), total bilirubin (TBIL), and total cholesterol (TC).

### 2.3. Histopathology Analysis

The left liver lobe was paraffin-embedded, sectioned at 4 μM, and processed for SHG/TPEF imaging, using hematoxylin and eosin, Sirius red, and reticulin staining. All samples were independently and blindly evaluated by two expert pathologists, and consensus scores were assigned based on the MASH CRN scoring system [20,21]. Steatosis was graded from 0 to 3 (<5% to >66% hepatocyte involvement). Inflammation was defined as the presence of intralobular inflammatory foci containing at least five leukocytes and accompanied by disruption of hepatic plates or increased hepatocellular eosinophilia. Inflammation scores were determined by averaging the total number of inflammatory clusters across five fields in hematoxylin and eosin (H&E)-stained sections examined at 100x magnification (3.1 mm^2^ per field). The scoring scale was as follows: normal, 0 (<0.5 foci); slight, 1 (<0.5–1 foci); moderate, 2 (1–2 foci); and severe, 3 (>2 foci). Two images per liver section were captured at 100× magnification, and the mean value was recorded. Fibrosis was also evaluated subjectively to assess severity and distribution patterns (perisinusoidal, periportal, pericentral, or bridging). Fibrosis severity was scored as absent (0), mild (1), moderate (2), or severe (3).

### 2.4. Immunohistochemical Analysis

For immunohistochemical analysis, tissue sections were deparaffinized in xylene, rehydrated through a graded alcohol series, and subjected to microwave heating in 10 mM citrate buffer (pH 6.0) at 500 W for 2–5 min for antigen retrieval. After rinsing in Tris-buffered saline (pH 7.6), endogenous peroxidase activity was blocked using 3% H_2_O_2_. Sections were then incubated with primary antibodies overnight at 4 °C. Following washing, sections were incubated with the corresponding secondary antibody for 30 min at 37 °C, and the signal was visualized using 3,3′-diaminobenzidine. Counterstaining was performed with hematoxylin. The primary antibodies used were anti-CD34 (1:500; Servicebio, Wuhan, China) and anti-Ki67 (1:600; Servicebio).

### 2.5. AI Analysis of Hepatic Steatosis and Fibrosis in Tumor and Non-Tumor Tissue

HistoIndex’s Genesis^®^ system, an automated SHG/TPEF microscope (HistoIndex, Singapore), was used to image unstained formalin-fixed, paraffin-embedded liver sections. SHG signals were used to visualize collagen, and TPEF signals were used to visualize surrounding cellular structures. Tissue samples were illuminated with a 780 nm two-photon laser; with SHG and TPEF signals detected at 390 nm and 550 nm, respectively. Tile-based imaging was performed at 20× magnification, with each tile comprising 512 × 512 pixels and covering an area of 200 × 200 μm^2^; adjacent tiles were used to capture the entire tissue section. Tumor and non-tumor regions were delineated based on pathologist-annotated stained reference slides. After that, AI-based algorithms segmented each region into specific histological subregions, including central vein, portal tract, and perisinusoidal areas. Fibrosis and steatosis were quantified by extracting collagen-related SHG features and lipid vesicle-related TPEF features across the entire sample and within each subregion. This method provides an objective, reproducible, and fully quantitative evaluation of liver histopathology [19].

### 2.6. Statistical Analysis

Data are presented as mean ± standard deviation. For statistical analysis, we selected the appropriate analysis of variance model based on the comparison objectives: One-way ANOVA was used to compare various outcome measures (including body weight, tumor size, tumor number, biochemical indices, pathological scores, and AI-based indicators) within the same sex across different modeling weeks. Two-way ANOVA was applied to examine the independent main effects of sex and modeling week, as well as their interaction, on the aforementioned outcome measures. Partial eta squared (η^2^) was calculated as the effect size indicator. Spearman’s rank correlation analysis was conducted to assess the correlation between the steatosis and fibrosis data quantified from SHG/TPEF images and conventional histopathological scores. Statistical significance was defined as *p* < 0.05. All analyses were conducted using GraphPad Prism version 10.1.2 (Boston, MA USA).

## 3. Results

### 3.1. Differences in Tumor Burden and Biochemical Alterations

**Figure 1:**
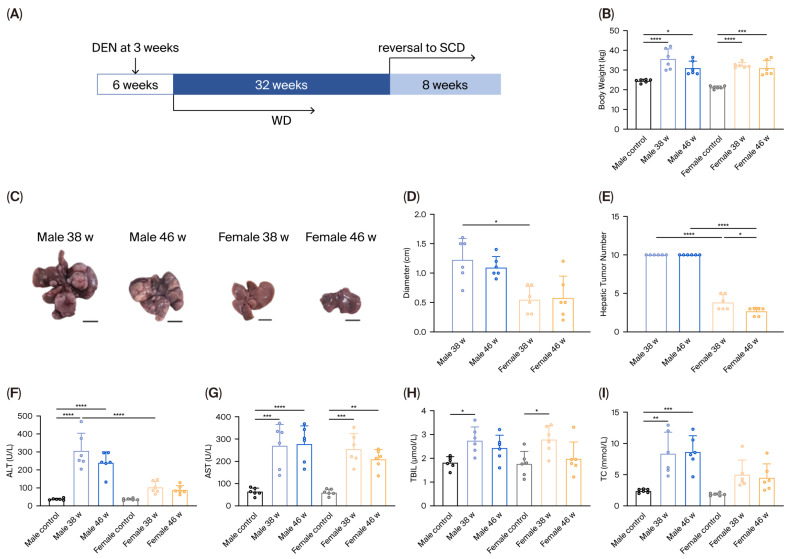
Tumor development in male and female mice treated with DEN and WD (**A**) Schematic overview of the MASH-associated HCC model in male and female mice. (**B**) Body weight changes in male and female mice over time. (**C**) Representative gross images of HCC nodules. Scale bar = 100 mm. (**D**) largest tumor size per mouse. (**E**) Tumor number per mouse. (**F**–**I**) Serum biochemical measurement after 38 weeks of WD feeding and after diet reversal, including (**F**) ALT, (**G**) AST, (**H**) TBIL, and (**I**) TC. Note: *, *p* < 0.05; **, *p* < 0.01; ***, *p* < 0.001; ****, *p* < 0.0001; DEN, diethylnitrosamine; WD, Western diet; SCD, stand chow diet; w, weeks; ALT, alanine aminotransferase; AST, aspartate aminotransferase; TBIL, total bilirubin; TC, total cholesterol; MASH, metabolic dysfunction-associated steatohepatitis.

After 38 weeks of WD feeding, both male and female mice showed considerable increases in body weight compared with controls (Figure 1B). However, male mice developed progressively larger hepatic tumors than female mice. Following an 8-week dietary reversal to normal chow, female mice exhibited a reduction in both tumor number and size; in contrast, male mice showed little to no improvement (Figure 1C–E).

Serum biochemical profiles corresponded with histological findings. Serum ALT, AST, and TBIL levels were significantly elevated in both male and female mice at 38 weeks relative to controls, indicating hepatocellular injury (Figure 1F–H). Additionally, TC levels were significantly higher in male mice compared with controls (*p* < 0.01) (Figure 1I). Notably, ALT levels were significantly higher in male mice than in female mice (*p* < 0.0001), suggesting more severe hepatocellular damage in males.

Together, these findings indicate that sex-related differences influence the extent of hepatic injury and tumor burden in the MASH-HCC model.

### 3.2. Histopathological Differences Between Tumor and Non-Tumor Tissue

**Figure 2:**
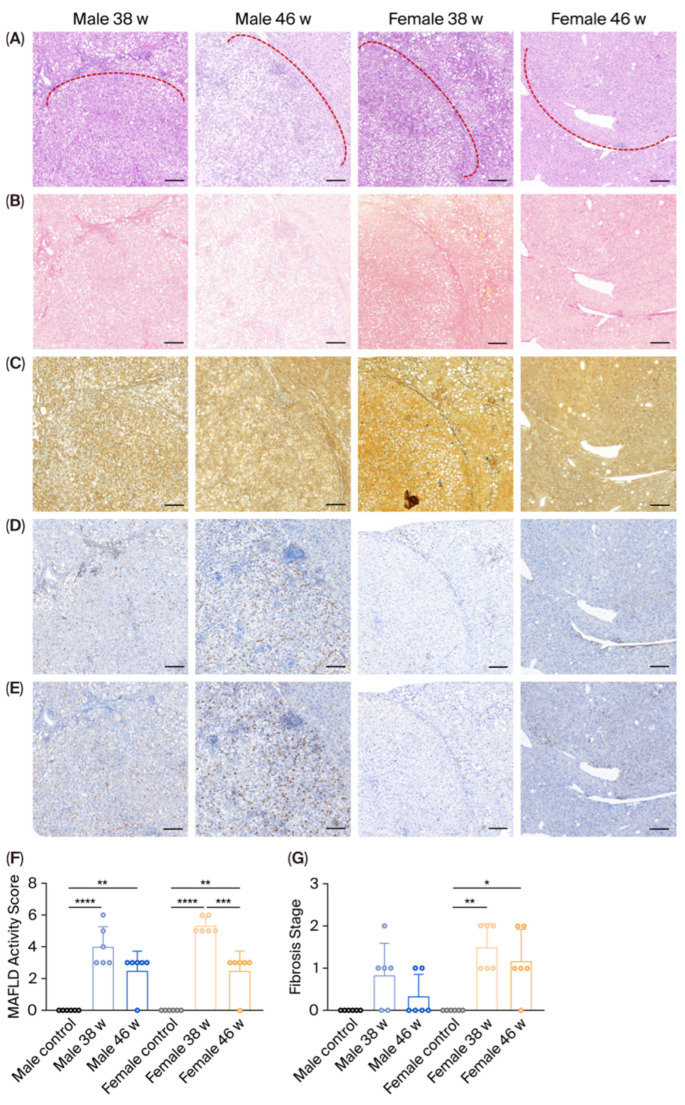
Representative images of (**A**) HE staining, (**B**) Sirius red staining, (**C**) reticulin staining, (**D**) CD34 and (**E**) Ki67 IHC, (**F**) MAFLD activity score, and (**G**) fibrosis stage in control, 38-w and 46-w male and female mice (100×). Note: *, *p* < 0.05; **, *p* < 0.01; ***, *p* < 0.001; ****, *p* < 0.0001; Red dotted lines indicate tumor margin; HE, hematoxylin and eosin; IHC, immunohistochemistry; WD, Western diet; w, week; MAFLD, Metabolic dysfunction-associated fatty liver disease; Bar: 200 μm.

Hematoxylin and eosin staining revealed marked steatosis within the tumor nodules, along with infiltration of inflammatory cells into the surrounding parenchyma. Sirius Red staining demonstrated prominent collagen deposition at the peritumoral boundaries; in contrast, reticulin staining showed a pronounced loss of reticular fibers within the tumor regions. Immunohistochemical staining for CD34 and Ki67 confirmed increased angiogenesis and proliferative activity, consistent with morphological features of HCC (Figure 2A–E).

Following withdrawal from the WD, both female and male mice exhibited a reduction in hepatic MAFLD activity score (MAS) and fibrosis in non-tumor regions, with females showing a particularly significant decrease in MASs (*p* < 0.001), as assessed by the MASH CRN scoring system (Figure 2F,G). These findings indicate that both male and female mice experienced histological regression after cessation of WD feeding.

### 3.3. AI-Assisted Assessment of Steatosis and Fibrosis Progression and Regression in Tumor and Non-Tumor Tissues

**Figure 3:**
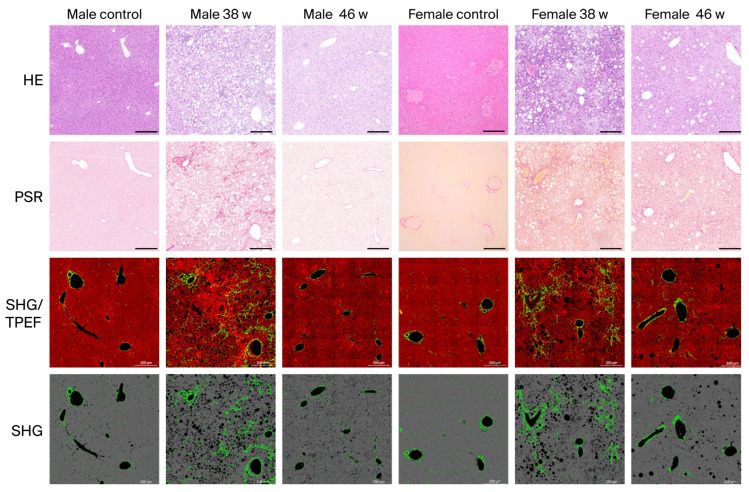
Representative images of H&E, PSR, SHG/TPEF, and SHG staining at control, 38 weeks, and 46 weeks in male and female mouse models. In the SHG/TPEF images, the red channel represents TPEF and the green channel represents SHG (collagen structure). The percentages of black fat vacuoles and their surrounding affected areas were quantified as steatosis. H&E, Hematoxylin and eosin; PSR, Sirius red staining; SHG/TPEF, second-harmonic generation/two-photon-excited fluorescence; w, weeks; WD, Western diet. Scale bar: 200 μm.

To objectively evaluate histological changes during the recovery phase, SHG/TPEF microscopy combined with AI–based image analysis was used to quantify steatosis and fibrosis. SHG/TPEF imaging demonstrated visible improvements in both lipid accumulation and collagen architecture following the return to a normal diet (Figure 3). Spearman’s correlation coefficient between AI and pathologist assessments reaches 0.85 for fibrosis-related parameters and 0.76 for steatosis-related parameters (Supplementary Tables S1 and S2). These high correlation values suggest that AI tool reliably captures the same histopathological features identified by human experts, thereby validating its utility for objective, reproducible quantification of steatosis and fibrosis changes.

**Figure 4:**
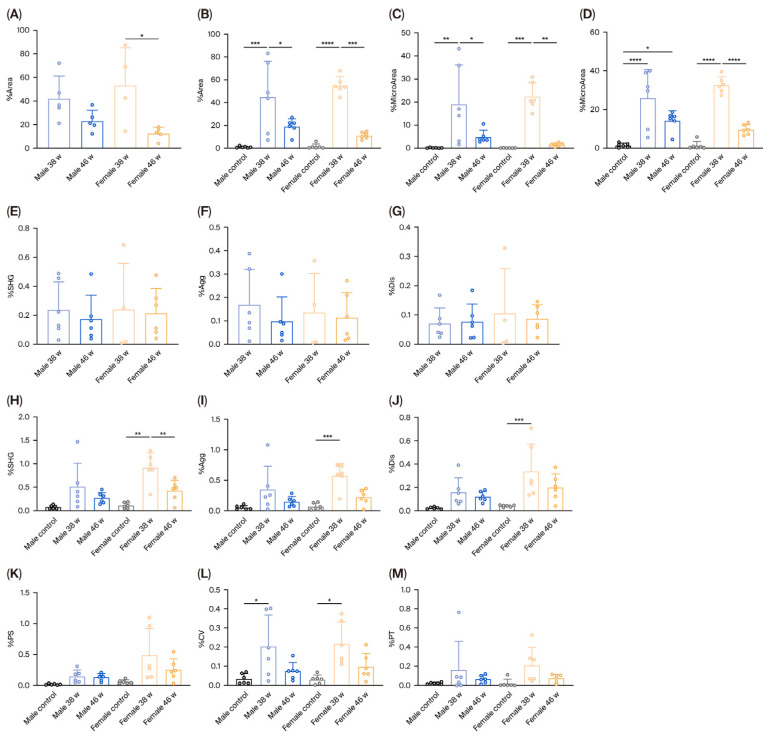
Changes in steatosis and fibrosis across tumoral and non-tumoral regions in male and female mice treated with DEN and WD. (**A**) Change in proportion of steatosis in tumor tissues; (**B**) Change in proportion of steatosis in non-tumor tissues; (**C**) Change in proportion of macrovesicular steatosis in non-tumor tissues; (**D**) Change in proportion of microvesicular steatosis in non-tumor tissues; (**E**) Change in proportion of total collagen (%SHG) in tumor tissues; (**F**) Change in proportion of aggregated collagen (%Agg) in tumor tissues; (**G**) Change in proportion of dispersed collagen (%Dis) in tumor tissues; (**H**) Change in proportion of total collagen (%SHG) in non-tumor tissues; (**I**) Change in proportion of aggregated collagen (%Agg) in non-tumor tissues; (**J**) Change in proportion of dispersed collagen (%Dis) in non-tumor tissues; (**K**) Change in proportion of collagen in the perisinusoidal area (%PS) of non-tumor tissues. (**L**) Change in proportion of collagen in the central vein area (%CV) of non-tumor tissues; (**M**) Change in proportion of collagen in the portal area (%PT) of non-tumor tissues; Note: *, *p* < 0.05; **, *p* < 0.01; ***, *p* < 0.001; ****, *p* < 0.0001; DEN, diethylnitrosamine; WD, Western diet.

AI-assisted quantitative analysis demonstrated that female mice exhibited a significant reduction in hepatic steatosis (%area) in both tumor and non-tumor regions after dietary recovery. This included reductions in total steatosis (%area), macrovesicular steatosis (%MacroArea), and microvesicular steatosis (%MicroArea). In male mice, steatosis (%area) decreased significantly only in non-tumor regions, primarily due to reductions in macrovesicular steatosis (%MacroArea) (*p* < 0.05). In contrast, steatosis within tumor regions declined; however, it did not reach statistical significance (*p* > 0.05) (Figure 4A–D).

In the MASH model, hepatic fat accumulation increases liver volume, which may affect the quantitative accuracy of fibrosis measurements. To objectively evaluate changes in fibrosis, a lipid droplet steatosis correction algorithm was applied to non-tumoral regions to exclude areas affected by steatosis, thereby eliminating its confounding impact on fibrosis quantification [22]. Under normal conditions, all collagen-related indicators in both male and female mice (%SHG: total collagen proportion in whole liver tissue; %Agg: aggregated collagen proportion; %Dis: dispersed collagen proportion) were low, reflecting minimal deposition across the central vein, portal tract, and perisinusoidal regions, consistent with healthy liver histology. To better reflect the biological significance of the observed differences in collagen-related parameters, we have supplemented the effect size estimates (partial η^2^) for all key indicators, including %SHG and %Agg (Supplementary Table S3), which showed that the grouping factor (sex × treatment time) exerted a statistically highly significant effect on (*p* < 0.01). After 38 weeks of DEN + WD treatment, all collagen parameters (%SHG, %Agg, and %Dis) were higher in female mice than in male mice at non-tumoral regions; however, differences were not statistically significant, suggesting more extensive multiregional collagen deposition in females. Eight weeks after resuming a normal diet, all collagen indicators declined in both sexes compared to those at the 38-week timepoint, differences were not statistically significant (Figure 4).

Collectively, these AI-based quantitative results demonstrate sex-dependent differences in hepatic recovery trajectories. Female mice exhibited a significant regression of steatosis during the recovery period after WD feeding, providing objective evidence of sex-specific hepatic plasticity in MASH-associated hepatocarcinogenesis.

## 4. Discussion

A DEN-WD-induced murine model combined with AI-enhanced SHG/TPEF imaging revealed sexual dimorphism in the progression and regression of MAFLD-HCC. Our findings demonstrate that male mice develop a higher tumor burden; in contrast, female mice exhibit a favorable response to dietary intervention, with greater reductions in tumor number and hepatic steatosis during the recovery phase. The results highlight the importance of considering biological sex in preclinical modeling insights deepen our understanding of sex-specific disparities in MAFLD-HCC.

Human data and animal studies have shown significant sex-specific differences in the prevalence of MAFLD and the associated risk of HCC. MAFLD incidence and prevalence are higher in men than in premenopausal women [9]. Yin et al. [23] conducted a meta-analysis demonstrating that male patients with non-alcoholic fatty liver disease have a significantly higher risk of developing HCC compared with female patients. In MAFLD mouse models, ALT and aspartate aminotransferase levels were significantly elevated in male mice fed a high-fat and choline-deficient diet compared to females [24]. Our results are consistent, showing a heightened tumor burden and elevated ALT levels in male mice with MASH-driven HCC. Previous studies attribute this disparity to hormonal, metabolic, and inflammatory differences between the sexes [24]. Androgen receptor signaling can promote malignant transformation by upregulating proinflammatory and proliferative genes [25], and activation of the sex-determining region Y contributes to male-specific hepatocarcinogenesis [26]. Conversely, the relative protection in female mice is largely attributed to estrogen [27,28,29]. Estradiol signaling protects female ApoE KO Mice against WD-Induced MASH by improving mitochondrial function [27], highlighting the relevance of our model in recapitulating this fundamental clinical disparity. Male-predominant factors, such as androgen signaling, exacerbate liver injury and drive hepatocarcinogenesis.

Notably, our study uncovered a critical divergence in the capacity for disease regression between sexes, an area less explored in previous research. Although the protective role of the female sex in disease initiation is recognized, its influence on recovery is less understood. Upon dietary recovery, female mice demonstrated a significantly greater reduction in tumor number and a resolution of hepatic steatosis in non-tumor regions compared with male mice. Traditional histological scoring systems are semi-quantitative and subject to interobserver variability; in contrast, integrating SHG/TPEF microscopy with AI-based morphometric analysis provides an unbiased and reproducible platform for quantifying microstructural changes in steatosis. Our AI-assisted quantification revealed a marked reduction in lipid vacuolation in female mice, corroborating these biological effects. These observations emphasize that sex hormones influence not only disease initiation but also the reversibility of MAFLD-related injury. The female liver demonstrate a greater capacity to adapt to varying dietary conditions and recover from metabolic stress compared to the male liver, efficiently reversing lipid accumulation once the metabolic stressor (WD/fructose) is removed. Estrogen signaling via ERα is a likely mechanism, enhancing fatty acid β-oxidation and suppressing lipogenic gene expression [28]. In contrast, the male liver may remain in a state of chronic inflammation and insulin resistance, limiting its ability to mobilize stored lipids and resulting in only marginal improvement. This disparity in recovery potential provides a plausible biological basis for variable patient responses to lifestyle interventions.

AI-quantified analysis indicated only moderate improvement of fibrosis in both sexes in non-tumor regions, unlike steatosis, which showed more pronounced recovery. These findings align with clinical observations [30,31]. In a randomized controlled trial of 71 patients who are overweight with NASH cirrhosis, semaglutide considerably reduced liver enzymes and hepatic fat; however, it did not induce significant improvement in fibrosis [30]. These results suggest that female mice have an advantage in collagen reversal and are more likely to return to baseline healthy levels after dietary intervention. Fibrosis resolution is a slow process involving extracellular matrix degradation and hepatic stellate cell inactivation [32,33,34,35,36]; the 8-week recovery period may have been insufficient for substantial regression. Nevertheless, SHG/TPEF imaging objectively captured these subtle improvements, highlighting its utility for dynamically monitoring fibrotic remodeling.

This study has some limitations. First, the use of a single genetic background (C57BL/6J) and a single hepatocarcinogenesis model (DEN + WD) may limit generalizability to other strains or etiologies. Future multi-center studies with expanded sample sizes, inclusion of additional mouse strains (including diverse MAFLD-HCC models), and validation across different experimental settings will be essential to confirm and extend these findings. Furthermore, we did not perform mechanistic analyses of sex hormone signaling or immune microenvironmental changes that could underlie the observed dimorphism. Future studies should incorporate hormonal manipulation, ovariectomy/castration models, and transcriptomic profiling to dissect the molecular basis of sex-dependent progression and regression of MAFLD-HCC. Thirdly, while the 8-week recovery period provides valuable insight into early regression dynamics, it remains unclear whether the observed improvements—particularly in fibrosis resolution—are sustained over time. Extended follow-up would better model clinical remission trajectories.

## 5. Conclusions

In summary, this study demonstrates sex-specific differences in the natural history and recovery of MAFLD-HCC, female mice showing enhanced metabolic and histological recovery following dietary normalization. This study provides valuable insights into the sexual dimorphism observed in MAFLD-related HCC and highlights the necessity of considering sex as a biological variable in preclinical research, which could lead to tailored therapeutic strategies for both sexes in clinical settings. It is the first to longitudinally assess steatosis, and fibrosis in both tumor and non-tumor liver regions, enabling high-resolution, quantitative insights into sexual dimorphism in MAFLD–HCC dynamics with AI-assisted SHG/TPEF imaging. AI-enhanced digital pathology offers a reproducible and translational tool for monitoring liver disease dynamics and identifying sex-specific regression patterns, which may inform personalized therapeutic strategies and enhance the predictive value of animal models.

## Data Availability

The raw data supporting the conclusions of this article will be made available by corresponding author (F.L.) on request.

## Supplementary Materials

The following supporting information can be downloaded at: https://www.mdpi.com/article/10.3390/diagnostics16020234/s1, Table S1: Correlation Analysis between Key Fibrosis Parameters and Fibrosis stages; Table S2: Correlation Analysis between Key Steatosis Parameters and Steatosis grades; Table S3: Effect Size Results of collagen-related Parameters.

## Abbreviations

The following abbreviations are used in this manuscript:

AI	artificial intelligence
MAFLD	metabolic dysfunction-associated fatty liver disease
HCC	hepatocellular carcinoma
MASH	metabolic dysfunction-associated steatohepatitis
DEN	diethylnitrosamine
WD	Western diet
SHG/TPEF	second harmonic generation/two-photon excitation fluorescence
ALT	alanine aminotransferase
AST	aspartate aminotransferase
TBIL	total bilirubin
TC	total cholesterol
