# Supplementary material for: Use of Artificial Intelligence-Assisted Histopathology for Evaluation of Sex-Specific Progression and Regression of Hepatocellular Carcinoma Related to Metabolic Dysfunction-Associated Fatty Liver Disease
Source: Diagnostics (Basel). 2026 Jan 11;16(2):234. doi: 10.3390/diagnostics16020234 (PMC12840184; doi:10.3390/diagnostics16020234)
Supplement: Supplementary file 1 [file diagnostics-16-00234-s001.zip › diagnostics-4061194-supplementary.pdf]

Supplementary Tables

Supplementary Table S1. Correlation Analysis between Key Fibrosis Parameters and Fibrosis stages

		%SHG	%Agg	%Dis	#Str	%CV	%PT	%PS
Fibrosis	correlation coefficient	0.852	0.851	0.820	0.817	0.768	0.634	0.811
	p value	<0.001	<0.001	<0.001	<0.001	<0.001	<0.001	<0.001

Supplementary Table S2. Correlation Analysis between Key Steatosis Parameters and Steatosis grades

		%Area	%MacroArea	%MicroArea
steatosis	correlation coefficient	0.753	0.756	0.742
	p value	<0.001	<0.001	<0.001

Supplementary Table S3. Effect Size Results of collagen-related Parameters

Indicator	partial η^2 value	p-value
%SHG	0.577	<0.001
%Agg	0.528	<0.001
%Dis	0.486	<0.01
#Str	0.469	<0.01
